# Discovery of a 240 million year old nematode parasite egg in a cynodont coprolite sheds light on the early origin of pinworms in vertebrates

**DOI:** 10.1186/s13071-014-0486-6

**Published:** 2014-11-13

**Authors:** Jean-Pierre Hugot, Scott L Gardner, Victor Borba, Priscilla Araujo, Daniela Leles, Átila Augusto Stock Da-Rosa, Juliana Dutra, Luiz Fernando Ferreira, Adauto Araújo

**Affiliations:** Museum National d’Histoire Naturelle, ISYEB, UMR 7205 CNRS, 55, rue Buffon, 75231 Paris, Cedex 05 France; Harold W Manter Laboratory of Parasitology, University of Nebraska State Museum and School of Biological Sciences, University of Nebraska–Lincoln, W529 Nebraska Hall, Lincoln, NB 685880514 USA; Escola Nacional de Saúde Pública Sergio Arouca, Fundação Oswaldo Cruz, Rua Leopoldo Bulhões 1480, 21041-210 Rio de Janeiro, Brazil; Departamento de Microbiologia e Parasitologia, Instituto Biomédico, Universidade Federal Fluminense, Rua Prof. Ernani Pires de Melo, 101, 24210-130 Niterói, Rio de Janeiro Brazil; Universidade Federal de Santa Maria, Departamento de Geociências, Laboratório de Estratigrafia e Paleobiologia, Campus Camobi, 97105-900 Santa Maria, Rio Grande do Sul Brazil

**Keywords:** Brazil, Cynodont, Coprolite, Haplodiploid, Heteroxynematidae, Oxyurida, Paleoparasitology, Pinworm, Upper Triassic

## Abstract

**Background:**

We report the discovery of a nematode parasite egg (Nemata: Oxyurida) from a coprolite closely associated with the remains of several species of Cynodontia, dated to 240 million years old. This finding is particularly significant because this is the oldest record of an oxyurid nematode yet discovered, and because the cynodonts are considered a stem-group of the mammals.

**Methods:**

We extracted material from a fully mineralized coprolite by both scraping the surface, and removing fragments from its interior with clean dental instruments used a single time. A single drop of glycerol from a new vial was added as a clearing reagent. Each slide was sealed with wax and examined with an optical microscope at 100× to 400× magnification.

**Results:**

From one coprolite, 550 slides were examined; from 275 of these slides, sediment was examined that was scraped from the surface of the coprolite, and from the other 275 slides, material was examined that was extracted from the interior of the coprolite. All microscopic structures encountered were photographed, measured, and identified when possible.

**Conclusions:**

From the coprolite examined, we discovered an egg representing a new species of pinworm that, based on the egg structure, clearly places it in the family Heteroxynematidae. Nematodes of the order Oxyurida have very constrained life-histories, occurring only in animals that are not strictly carnivorous and also ingest large amounts of plant material. This fact enabled us to determine which species of cynodont, from several collected at the site in Brazil, are most likely the depositors of the coprolite, and therefore were the putative host of the parasite.

## Background

The field of paleoparasitology has developed rapidly since its inception in the early 20th century up to the present time. Fossil parasites of both plants and animals have been found from a broad geological time-spectrum, ranging from the Holocene as far back as the lower Cambrian (over 500 million years) [1–3]. Studies of parasites in paleo-faunas can provide firm data on ages of fossilized organisms and allow establishment of both dates of origin and diversification for host-parasite associations, including groups of parasites associated with extinct vertebrates [4,5]. Coprolites can be important sources of data because the study of fossilized fecal material provides simultaneous information on both host and parasite enabling a better understanding of their ecological relationships [4–7].

Herein, we describe the egg of a nematode parasite (Nemata: Oxyurida) discovered in a single coprolite associated with species of primitive proto-mammals of the Class Cynodontia that is estimated to be about 240 million years old (MYO). The discovery is particularly significant for two reasons that include: a) This finding reports the most ancient pinworm yet discovered, and b) the host group, the cynodonts, are part of the stem group of the lineage of early vertebrates that includes the mammals.

All pinworms in the order Oxyurida have a very specific ecological/life-history trait, which is that almost all known hosts of these nematodes have a portion of their gut that acts as a cellulose decomposition chamber (via anaerobic bacterial/protistan digestion). This allows us to speculate as to which species of cynodont, from several that were recorded at the collection site, is most probably the depositor of the coprolite and, therefore, the host of the new parasite. Because of this finding, we can speculate as to the presence of Oxyurida in other extinct lineages of vertebrates.

## Methods

We extracted material from a single, fully mineralized coprolite by both scraping the surface, and removing fragments from the interior with clean dental instruments used a single time for each sample. Material extracted from each was examined separately. The material obtained was placed in EppendorfTM tubes and labeled. The material was then dissociated in this tube with a solution of 10% hydrochloric acid (HCl) and double distilled water (v/v) and immediately washed with distilled water until a neutral pH was achieved. Slides were prepared by placing a single drop of sediment from the material that was dissociated in HCl on a depression slide. To each slide, a single drop of glycerol from a new vial was added as a clearing reagent. The slide was sealed with wax and examined with an optical microscope at 100× to 400× magnification. From one coprolite, 550 slides were examined; from 275 of these slides, sediment was examined that was scraped from the surface of the coprolite, and from the other 275 slides, material was examined that was extracted from the interior of the coprolite. All microscopic structures encountered were photographed, measured, and identified when possible.

## Results

**Figure 1:**
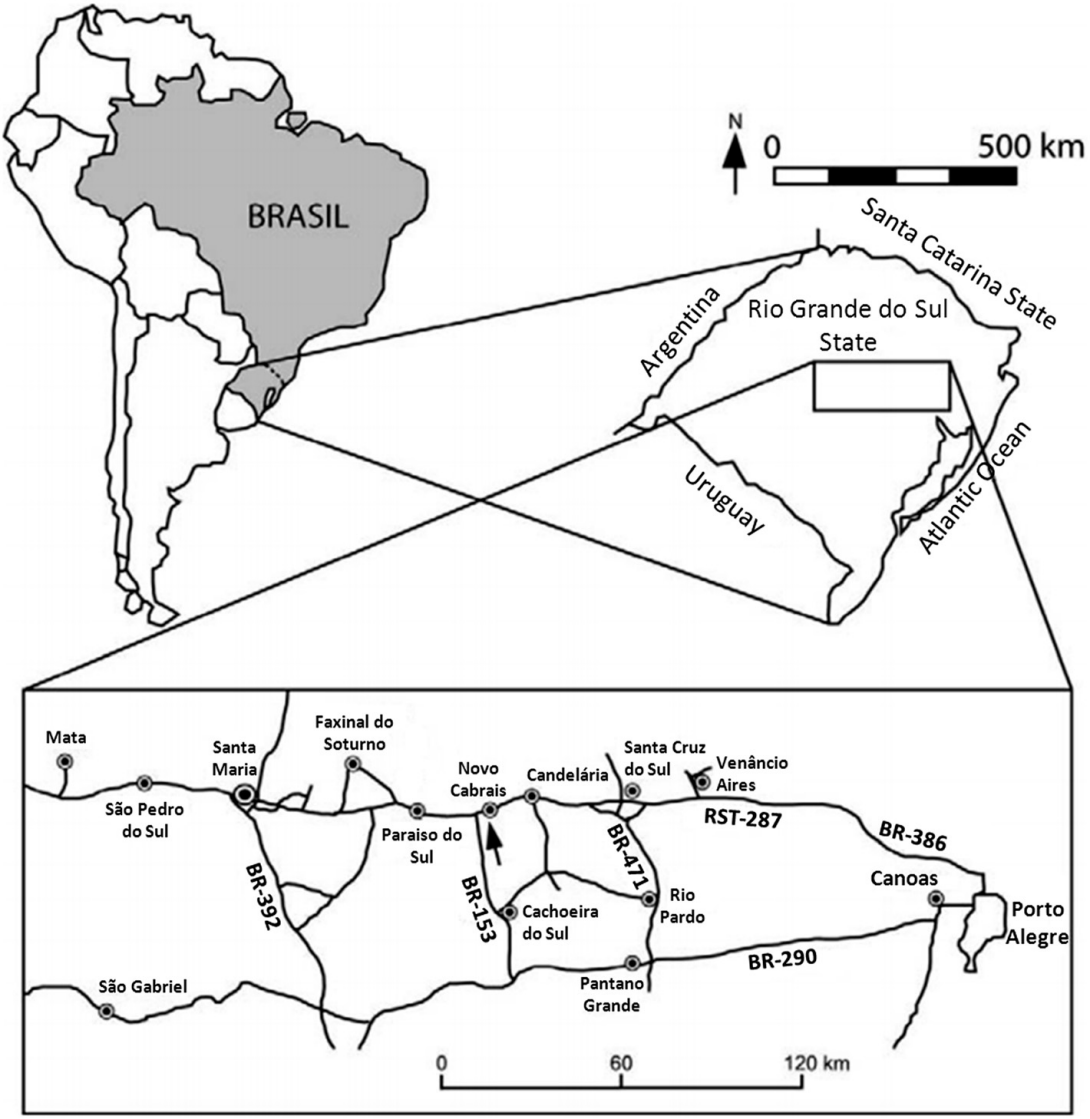
**Map showing the location of the Sítio Cortado site in Rio Grande do Sul State, Brazil where the specimens of the cynodonts and the coprolites were recovered.**

**Figure 2:**
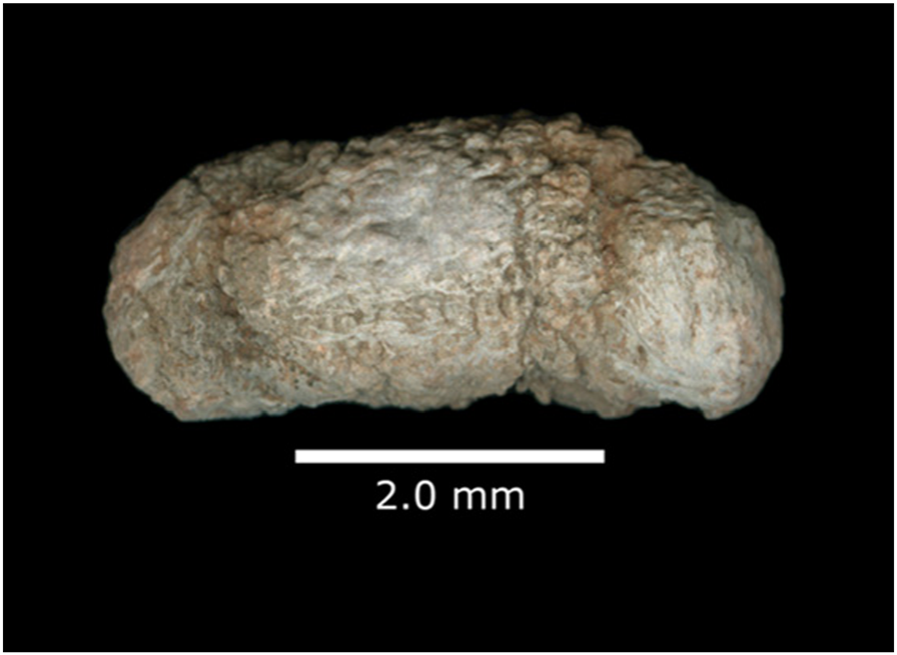
**Cynodont coprolite from the Upper Triassic, Sítio Cortado site, Rio Grande do Sul State, Brazil.**

The coprolite that we studied originated from excavations conducted at the Sítio Cortado site, Rio Grande do Sul State, Brazil [7–9] (Figure 1) and is completely permineralized and petrified with calcite (CaCO_3_). The coprolite was identified as fossilized feces that were derived from a cynodont because of its intimate association with abundant fossil remains of these animals at the site of collection. The single coprolite studied (Figure 2) was examined with a stereomicroscope to ascertain its texture and morphological features. During our work, nematode eggs representing two different species were found: An ascarid-like egg (previously described [9]) and the egg of a pinworm that we report and describe as a new species in the following.

### Taxonomic description

#### Paleoxyuris cockburni n. gen., n. sp.

**Figure 3:**
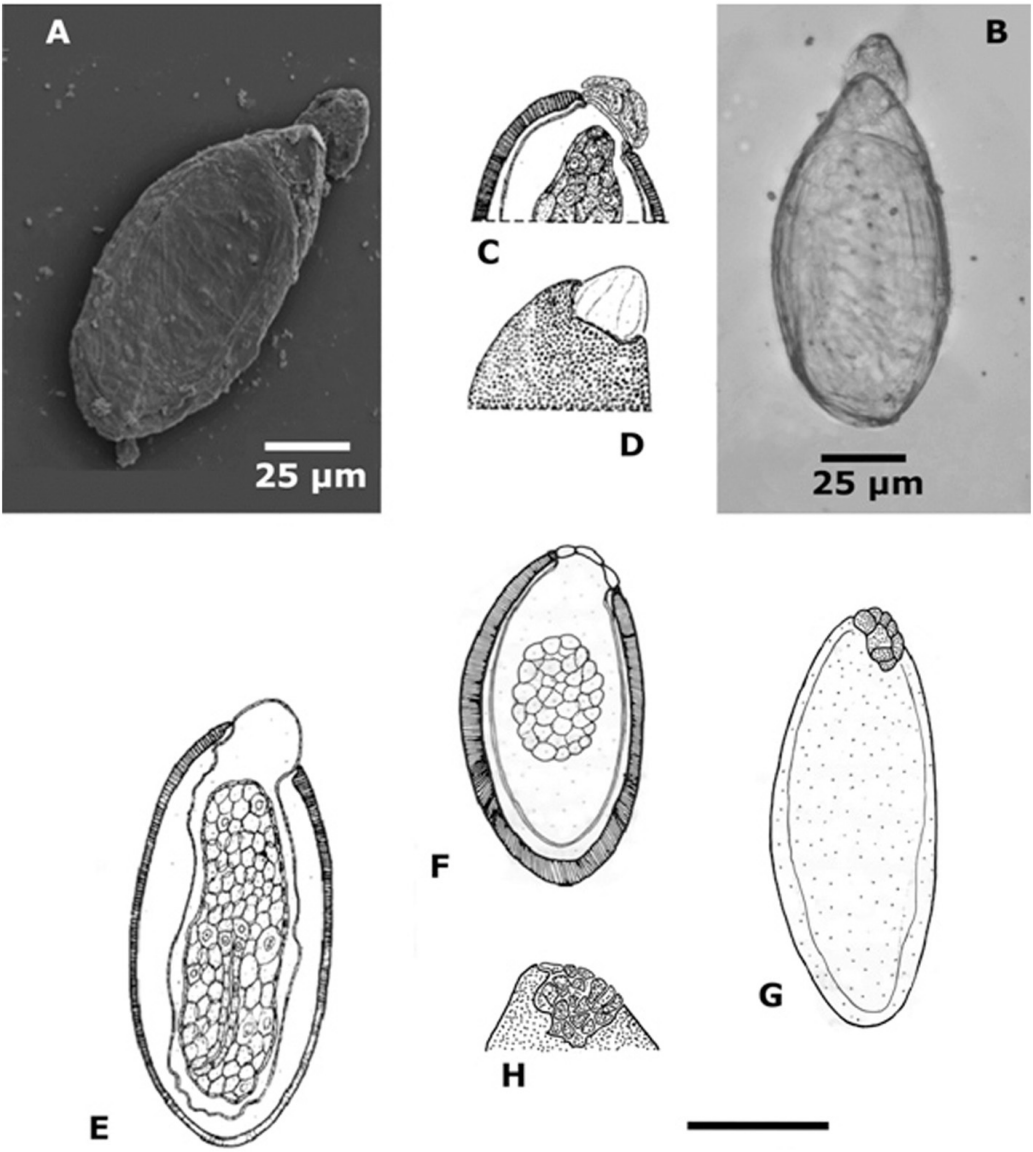
***Paleoxyuris cockburni*** **n. gen., n. sp. A**, egg from a coprolite (SEM microscopy); **B** *idem*, (light microscopy); *Syphaciella madagascariensis* Vassiliadès, 1970 egg: **C** and **D** detail of the operculum; **E**, optical cut. *Heteroxynema (Cavioxyura) viscaciae* Hugot and Sutton, [10] egg: **F**, optical cut; **G**, lateral view; **H**, detail of the operculum. Scale: E, F, G, 100 μm; **C**, **D**, **H**, 50 μm. [**C**, **D** and **E**, after Hugot [11]; **F**, **G** and **H**, after Hugot and Sutton [10]].

The single egg of this pinworm nematode is ellipsoidal, slightly flattened on one side, one extremity more rounded, two-layered, smooth-surfaced shell, measuring 113.51 × 77.08 μm. Embryonic mass visible, but amorphous; polar cap visible (Figure 3A).

*Phylum*: Nemata

*Order*: Oxyurida Railliet, 1916

*Family*: Heteroxynematidae (Skrjabin and Shikhobalova, 1948)

*Genus*: *Paleoxyuris* n. gen.

*Species*: *cockburni* n. sp.

*Paleoxyuris cockburni* n. gen., n. sp.

LSID (Zoobank): urn:lsid:zoobank.org:pub:49EFE6C7-860 F-436 F-8FE3-178A3F77C49B

*Etymology*: “paleo,” ancient, archaic, and “oxyuris”. The species name is a tribute to Aidan Cockburn, founder of the Paleopathology Association.

*Type locality*: In a cynodont coprolite, Upper Triassic, Rio Grande do Sul State, Brazil. Latitude longitude and date collected. (S29 °30′-S29 °45′–W53 °15′-52 °45′, collected in 2007).

*Type specimen*: Coprolite collected by Átila Da-Rosa in the field and deposited in the collection of coprolites and archeological and paleontological remains, Paleoparasitology Laboratory, Sergio Arouca National School of Public Health, Oswaldo Cruz Foundation, Rio de Janeiro, Brazil; slide and digitized image #A-1578B.

#### Differential diagnosis

In an extensive review of the taxonomic characteristics of the Nemata, Chitwood and Chitwood [12] demonstrated that the morphology of the eggs of nematodes provide reliable and robust information for the diagnosis of the taxonomic groups at level of families, genera, and sometimes species. Most known species of Oxyurida have eggs with an operculum, or with a lateral cap; a few species have a polar cap (as seen in our specimen) and these (with polar or terminal caps) are all classified in the family Heteroxynematidae Skrjabin and Shikhobalova, 1948. A comparison of the egg of *P. cockburni* n. sp. with all the eggs of the Oxyurida pictured in Chitwood and Chitwood [12] shows that it is very similar in size and shape to the eggs of several species of the genera *Heteroxynema* Hall, 1916 (Figure 3F,G and H) and *Syphaciella* Mönnig, 1924 (Figure 3C,D and E). These characteristics enable us to place *Paleoxyuris* in the family Heteroxynematidae. Nematodes of the genus *Heteroxynema* are known parasites of both rodents and lagomorphs, species of *Syphaciella* (also in the Heteroxynematidae) are parasites of sand grouse of the genera *Syrrhaptes* and *Pterocles* (Pterocliformes: Pteroclidae) that are ground-nesting birds restricted to desert and semi-deserts of the Palearctic zoogeographic region. *Paleoxyuris cockburni* n. gen., n. sp. can be separated from the only other nematode parasite known from cynodonts collected from the same time stratum by the shape [9] with *P. cockburni* showing the typical pinworm shape while the other nematode is obviously an ascarid [9] with a completely different shape and structure.

## Discussion

### Pinworms as a natural group

**Figure 4:**
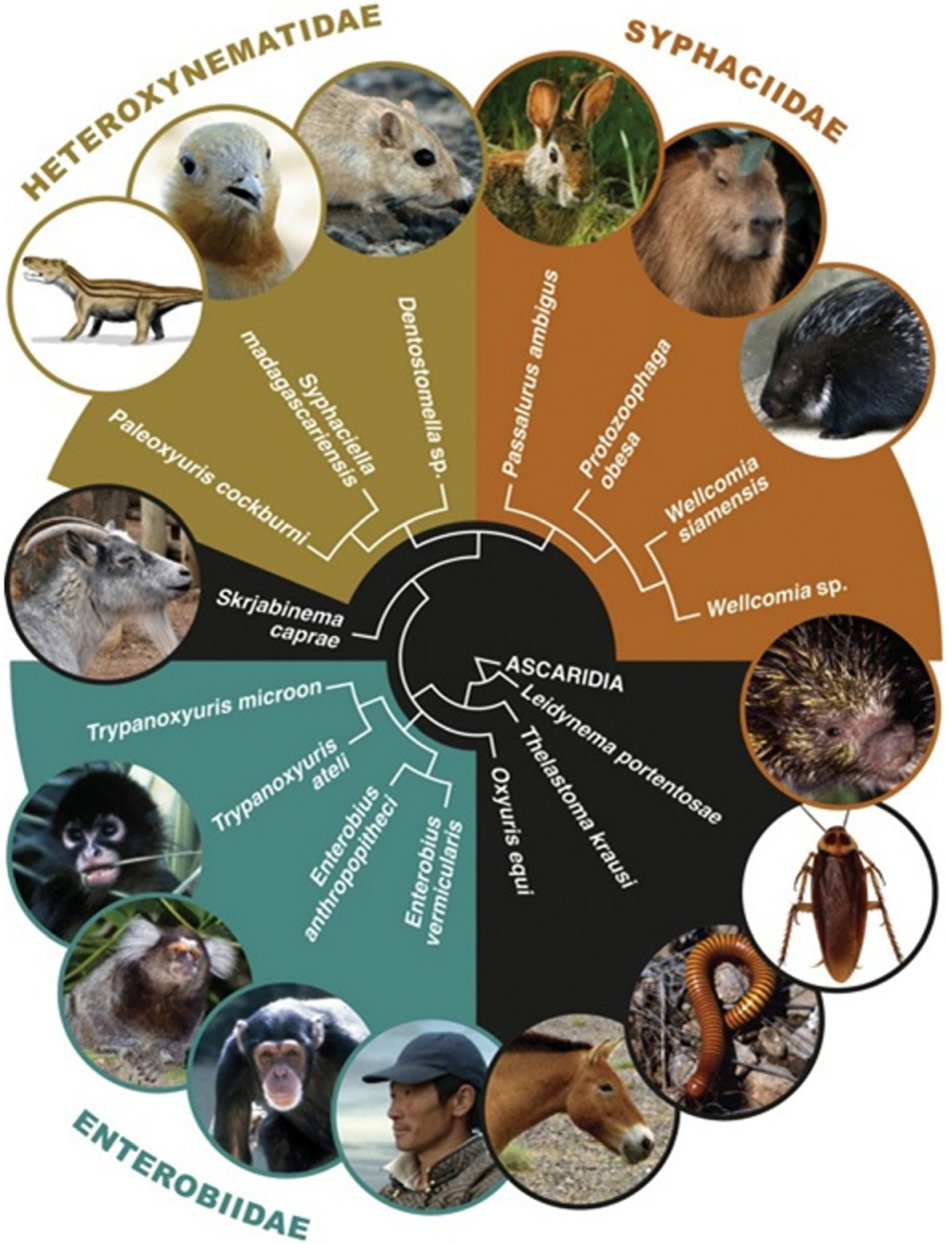
**Origin and classification of pinworm parasites: from arthropods to mammalian reptiles and humans.** The Oxyurida are parasites of both vertebrate and invertebrate with around 800 to 1000 species being described up to the present time. Few molecular analyses have been performed on the group as a whole, but whenever several species of pinworms were compared with other nematodes, they appear as a monophyletic sister-group of the Ascarida [14–16]. This tree summarizes the results of these different studies. It is remarkable that whatever the family, Heteroxynematidae or Syphaciidae, all the pinworm parasites of both rodents and lagomorphs always show a common origin. Based on the morphological characters of the egg, *Paleoxyuris cockbruni* n. gen., n. sp., a parasite of cynodonts in the family Traversodontidae, is classified in the Heteroxynematidae.

Within the Nemata, the Oxyurida is the only order with representative species occurring as parasites in both vertebrates and invertebrates (Figure 4). They are found in arthropods [12,13], fishes, birds, lizards, amphibians, and mammals [13]. Whatever their host group, pinworms feed on endosymbiotic bacteria and protists living in the digestive tract of their hosts and these parasites only occur in animals that digest relatively large quantities of cellulose. One question then arises: Did the Oxyurida have a single common origin or is the group polyphyletic, having originated more than once from ancestral nematode species. Several lines of evidence provide support of the Oxyurida as a natural or monophyletic group.

Recent studies using molecular data to define the phylogenetic relationships of the main groups of the phylum Nemata all agree in the placement of species of Oxyurida in a monophyletic group or clade [15–17] (Figure 4). All Oxyurida thus far investigated have a haplodiploid mode of reproduction: Unfertilized eggs give rise to haploid males and fertilized eggs always produce diploid females [18]. There is currently no evidence showing a reversal of this mode of reproduction in any taxa of the Nemata or other groups of organisms that manifest a haplodiploid mode of reproduction; this list includes, but is not restricted to, the Hymenoptera (ants, wasps, honey bees), Homoptera, and some mites (Acari: Mesostigmata). Data available from the phylogenetic databases of the Nemata, and the groups mentioned above, show clearly that once a species has made the transition from a diploid to a haplodiploid mode of reproduction, reversal back to a diploid system of sexual recombination is unlikely. We therefore interpret haplodiploidy in the Nemata as an ancient life-history character that probably originated simultaneously with the speciation event that gave rise to the Oxyurida [13].

### Host specificity and coevolution

Host specificity gauges the degree to which a parasite species is restricted to a particular host species without reference to phylogeny [18]. Pedersen *et al.* [19] showed that parasites using close contact transmission are primarily specific to single host species. Pinworms certainly belong to this category. In addition to having a direct life-cycle without involving an intermediate host, observations demonstrate that different behaviors of the hosts of pinworms facilitate direct-transmission of the parasite eggs thus enabling successive self-infections [20,21]. Common behaviors of host animals that may increase the probability of repetitive self-infections are: coprophagy (feeding on excrements) and caecotrophy (re-ingestion of fecal pellets naturally produced by digestion from the cecum).

Parasites with direct life-cycles occurring in hosts that manifest behaviors that increase the probability of self-infection appear to be able to maintain this association for long periods of time. At the same time these characteristics of both the host and parasite appear to decrease the probability that the parasite would switch to a new species of host. One evolutionary consequence of host specificity is a long-standing association between hosts and parasites through geological time. Darwin [22] first discussed this idea and afterwards, many authors have subsequently suggested that the phylogenetic relationships of highly host-specific parasites could provide valuable information on the evolutionary history of their hosts [6,23–28]. This defines the fields of Historical Ecology and Cophylogeny: The study of two or more associated groups of organisms sharing a common history of speciation, making possible phylogenetic conclusions. Accordingly, numerous studies have produced evidence for extreme host specificity and patterns of cophylogeny among mammals and their oxyurid parasites [27,28].

### Identification of the host

The Cynodontia, are therapsid vertebrates that first appeared in the Late Permian (approximately 260 Mya). The group includes modern mammals as well as their extinct ancestors and close relatives. Non-mammalian cynodonts spread throughout southern Gondwana and are represented by fossils from South America, Africa, India, and Antarctica. The non-mammalian cynodont fossils collected from the site from where the coprolite was identified belong to the species *Massetognathus ochagaviae* Barberena, [29]; *Chiniquodon theotonicus* Huene, [30]; *Traversodon stahleckeri* Huene, [30]; *Luangwa sudamericana* Abdala and Sá-Teixeira, [31]; and *Protheriodon estudianti* Bonaparte *et al*. [32]. *Massetognathus* and *Traversodon* were most abundant in the cenozone of the site [7,8].

Species of *Chiniquodon* and *Protheriodon* were carnivorous cynodonts, which lived during the early Late Triassic in South America. *Traversodon, Luangwa*, and *Massetognathus* all are members of family Traversodontidae, which includes fairly advanced, plant-eating, non-mammalian therapsids [8]. For instance, *Massetognathus* had the distinctive long snout of its cynodont relatives, with nipping incisors and fang-like canines, but its cheek teeth were flat- topped and covered with low ridges, which made them good for grinding stems, roots and other plant materials. Thus, the fauna associated with the coprolite is dominated by herbivorous or semi-herbivorous animals.

Species of animals (including humans and other primates) infected by pinworms always consume large quantities of cellulose, with no known exceptions, regardless of their phylogenetic affinities: vertebrate or arthropod. This feeding behavior may be considered necessary for the Oxyurida to be able to infect and survive in a host. Thus, the cynodonts that were herbivorous and were found at the collection site appear to be the most plausible sources of the coprolite from which we found the pinworm: *i.e.* one of the species identified in the Traversodontidae (Figure 4).

Species assigned to the Traversodontidae appear to have had a primarily Gondwanan distribution, with many species known from Africa and South America [8]. Battail [33] has hypothesized that they originated in what is now South America with subsequent diversification east into Africa and north into what is now Europe and eastern North America. We therefore consider representatives of the family Traversodontidae identified at the site of discovery of the coprolite, as the hosts of the new pinworm species described in this work. We also consider that the Traversodontidae are the primitive hosts of the Heteroxynematidae with a Gondwanan origin for both families (Figure 4).

### Did the dinosaurs have pinworms?

**Figure 5:**
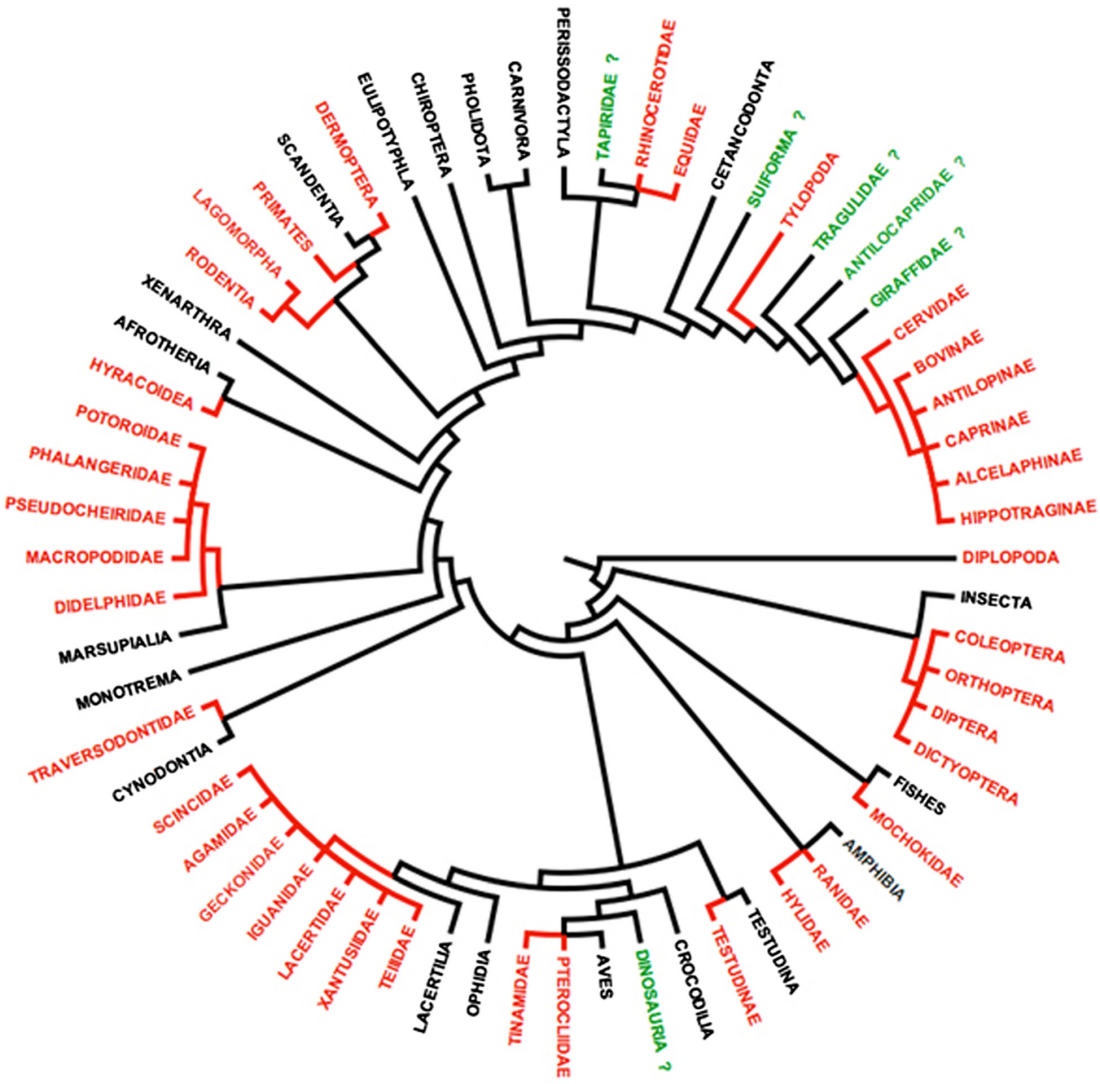
**Phylogenetic relationships of the animal groups parasitized by species of the order Oxyurida.** Most animal groups that consume large quantities of plant material (cellulose) are parasitized by pinworms (in red). Generally, these parasites are found in all the representatives of a family group over the complete geographic distribution of the family. Different pinworm species identified in one host group are morphologically very similar, indicating host-parasite cospeciation. However, some groups have not been shown to harbor oxyurids (in green and indicated by a question mark) even though the feeding habits and digestive tract seem appropriate. The question also can be asked regarding the presence of pinworms in herbivorous dinosaurs.

The discovery of an egg of a nematode of the order Oxyurida in the Cynodontia shows that this host-pinworm parasitic association existed as far back as the transition point of the reptile-mammal phylogenetic divergence, approximately 240 million years ago. This finding shows that parasitism as an ecological trait originated at least as early as this group of cynodont infecting nematodes and that this group of nematodes has successfully been infecting herbivorous mammals (and birds to a lesser extent) to the present time. Most animal groups that contain herbivorous representatives are parasitized by pinworms (Figures 4 and 5). This begs the question of the existence of pinworms in extinct animal lineages having similar feeding behaviors: as all herbivorous vertebrate groups, including birds, are infected with pinworms, the probability for the extinct herbivorous dinosaurs to be unaffected may be considered to be low; especially considering the extreme variety and wide geographical distribution of the herbivorous lineages within the Dinosauria.

## Conclusion

The morphological characteristics of the egg of the pinworm parasite discussed herein (and an ascarid egg from the same coprolite [9]) were well-preserved for millions of years, enabling identification using routine methods of microscopy. This shows that, with appropriate techniques, additional fossil records of nematodes may be discovered. In the recent past, Dorris *et al.* [14] assumed that the absence of fossils made it impossible to date the stages of evolution in nematodes. We have discovered evidence of parasitism in extinct animals, providing precedent for additional studies in fossils dating back millions of years. It also shows that the pinworm lineage extends at least as far back as 240 million years, from at least as old as the mammal-like reptiles through evolutionary diversification to modern vertebrates including humans and other primates. Finally, the discovery of these parasites in a group of hosts that are the stem group that gave rise to the mammals expands the scope and range of potential comparative studies, which for the moment are limited to morphology but may soon expand into molecular investigations. Our study also highlights the need for additional combined molecular/morphological and field-based collections and studies that will improve the resolution of the phylogenetic relationships of the major families classified within the order Oxyurida.
